# Factors influencing the difficulty of laparoscopic cholecystectomy: A review

**DOI:** 10.6026/973206300220722

**Published:** 2026-02-28

**Authors:** Atul Kumar, Bhavesh Khandelwal, Shivani Sinha, Neelam R Charles

**Affiliations:** 1 Department of Surgery, Dr. Laxmi Narayan Pandey Government Medical College, Ratlam, Madhya Pradesh, India

**Keywords:** Laparoscopic, cholecystectomy, conversion, risk factors, gallbladder wall thickness, scoring systems

## Abstract

Laparoscopic cholecystectomy (LC) is the preferred treatment for symptomatic cholelithiasis, yet conversion to open surgery occurs in a notable proportion of cases due to factors such as dense adhesions, unclear anatomy, or intraoperative complications. Therefore, it is of interest to identify preoperative predictors of difficult LC to guide surgical planning. Across the included studies, factors consistently associated with conversion included gallbladder wall thickness greater than 4-5 mm, contracted gallbladder, acute cholecystitis, age over 60 years and male gender. Other factors such as diabetes mellitus and white blood cell count were not reliably predictive. Thus, we show the importance of structured preoperative assessment and support the use of predictive scoring systems to estimate surgical difficulty, optimize operative planning and improve patient outcomes.

## Background:

Laparoscopic cholecystectomy (LC) has become the gold standard for gallbladder removal due to its minimally invasive nature, leading to reduced postoperative pain, shorter hospital stays and quicker recovery times. However, the procedure's complexity can vary significantly among patients, with certain cases posing challenges that increase the risk of complications and conversion to open surgery [1]. Understanding the predictors of difficult LC is crucial for preoperative assessment, surgical planning and patient counseling. Several preoperative factors have been identified as indicators of potential surgical difficulty. These include advanced age, male gender, obesity and a history of acute cholecystitis [2]. Additionally, ultrasonographic findings such as thickened gallbladder walls, impacted stones and pericholecystic fluid collections have been associated with increased surgical complexity [3]. Intraoperative factors also play a significant role in determining the ease of the procedure. The presence of adhesions, difficulty in identifying the Calot's triangle and the need for prolonged operative time are indicative of challenging cases [4]. To quantify these variables and predict surgical difficulty, various scoring systems have been developed. These models integrate clinical, biochemical, radiological and intraoperative parameters to stratify patients based on their risk of experiencing a difficult LC [5]. Recognizing patients at high risk for difficult LC allows for better surgical planning, appropriate resource allocation and informed patient discussions regarding potential outcomes and complications. Therefore, it is of interest to enhance patient safety and optimize surgical success rates.

## Overview:

## Sequential evaluation of predictors of difficult LC:

Across the literature, predictors of difficult laparoscopic cholecystectomy (LC) have been evaluated in a logical sequence beginning with patient-related clinical factors, followed by radiological and biochemical parameters, and finally intra-operative correlates and validated scoring systems. Initial clinical assessment consistently identifies increasing age, male sex, elevated body mass index (BMI), and a previous history of acute cholecystitis (AC) as major determinants of operative difficulty. In recent prospective work, Garg *et al.* (2025) [6] demonstrated that a BMI greater than 27 kg/m^2^ and gallbladder (GB) wall thickness exceeding 4 mm were significantly associated with difficult LC. Building on this, Teja *et al.* (2025) [7] sequentially showed that, in addition to these clinical factors, a common bile duct (CBD) diameter greater than 6 mm and impacted Hartmann's pouch stones independently predicted difficulty, reporting a conversion rate of 9.5%. Similar stepwise associations between clinical history and ultrasonographic findings were reported by Stoica *et al.* (2024) [8] and Toppo *et al.* (2024) [9], both of whom achieved sensitivities exceeding 80% when these parameters were combined. Following identification of individual predictors, several studies progressed toward the development of structured preoperative scoring systems.

## Role of prior acute cholecystitis and recurrent inflammation:

Teerawiwatchai *et al.* (2024) [10] integrated age, male sex, prior abdominal surgery, GB wall thickness greater than 3 mm, and multiple stones into a composite model, achieving an AUC of 0.89. Subsequently, Manjunath *et al.* (2020) [11] extended this sequential framework by emphasizing intra-operative findings such as dense adhesions, GB distension, and the presence of bile or pus, resulting in excellent predictive accuracy with an AUROC of 0.992. Radiological evaluation, particularly ultrasonography, represents the next critical step in preoperative risk stratification. Across multiple studies, GB wall thickening greater than 4 mm, pericholecystic fluid, impacted neck or Hartmann's pouch stones, GB distension or contraction, large stones exceeding 2 cm, and CBD dilation were repeatedly identified as strong predictors of difficulty. These findings were consistently reported by Bhandari *et al.* (2021) [12]. Beyond clinical and radiological assessment, biochemical and inflammatory markers have been evaluated as adjunctive predictors. De Buono *et al.* (2021) [13] demonstrated that elevated white blood cell count, AST, and fibrinogen levels independently predicted difficult LC. Nassar *et al.* (2020) [14] evaluated preoperative clinical and ultrasonographic predictors of difficult laparoscopic cholecystectomy in a retrospective cohort, finding that age >40, ASA ≥2, prior ERCP, GB wall >3 mm, and CBD >6 mm were significantly associated with increased operative difficulty. Intra-operative surrogates of difficulty, including prolonged operative time, bile spillage, ductal or arterial anomalies, and dense adhesions, were subsequently shown to correlate closely with these preoperative indicators in studies by Putra *et al.* (2022) [15].

## Radiological predictors (Ultrasonography-Based Risk Factors):

Bhondave *et al.* (2017) [16] prospectively evaluated clinical and ultrasonographic predictors of difficult laparoscopic cholecystectomy (LC), identifying BMI, prior acute cholecystitis (AC), gallbladder (GB) wall thickness, distension, impacted stones, and total leukocyte count as significant determinants. Kulkarni *et al.* (2018) [17] reported that male sex, recent biliary pain, jaundice, diabetes mellitus, palpable GB, number and size of stones, wall thickening, and fatty liver increased operative difficulty. Mannivannan *et al.* (2017) [18] found that a distended or contracted GB, wall thickness greater than 3 mm, large stones, and pericholecystic fluid were key predictors of conversion. Parmar *et al.* (2018) [19] demonstrated that age, sex, history of AC, fever, tenderness, elevated amylase, LDH, leukocytosis, CBD diameter, and GB wall thickness greater than 4 mm correlated with difficult LC. Vivek *et al.* (2014) [20] developed a scoring system including age, sex, BMI >30, previous AC or surgery, post-ERCP status, deranged liver function tests, GB contracture, and multiple stones, which reliably predicted difficult LC. Veerank *et al.* (2018) [21] analyzed clinical, radiological, and intra-operative parameters, confirming that GB wall thickness, impacted stones, pericholecystic collection, and male sex were strong predictors. Ary Wibowo *et al.* (2022) [22] showed that preoperative factors such as age, male sex, prior AC, BMI, palpable GB, scars, wall thickness >4 mm, and leukocytosis could stratify LC difficulty using a scoring system.

## Predictors Related to Biliary Obstruction (CBD Dilatation, ERCP, Stones):

Bouarfa *et al.* (2011) [23] retrospectively demonstrated that age, male sex, high BMI, prior surgery, and wall inflammation predicted difficult LC with approximately 83% accuracy. Randhawa *et al.* (2009) [24] highlighted BMI >27.5 kg/m^2^, prior AC, palpable GB, wall thickening above 4 mm, impacted stones, and pericholecystic collection as preoperative predictors, achieving a positive predictive value of 92%. Lipman *et al.* (2007) [25] reported that male sex, age >60 years, diabetes mellitus, ASA ≥3, leukocytosis, hypoalbuminemia, and pericholecystic fluid were strongly associated with conversion. Giger *et al.* (2006) [26] in a large cohort confirmed male sex, BMI >30, and ASA III/IV as dominant risk factors for difficult LC. Nachnani *et al.* (2005) [27] found that BMI >30, male sex, prior AC or pancreatitis, and previous upper-abdominal surgery were predictive of conversion. Lal *et al.* (2002) [28] demonstrated that GB wall >4 mm, contracted GB, impacted neck stones, and CBD stones were associated with longer operative time, bile spillage, and higher conversion rates. Kama *et al.* (2001) [29] reported that age >60, male sex, tenderness, prior upper-abdominal surgery, wall thickening, and AC diagnosis correlated with higher conversion rates and operative difficulty. Alponat *et al.* (1997) [30] showed that age, sex, obesity, disease duration, prior surgery, fever, leukocytosis, deranged liver function tests, and wall thickening were significant predictors of conversion. Stanisic *et al.* (2014) [31] identified male sex, age >65, prolonged disease, prior surgery, wall thickness >4 mm, stones >2 cm, pericholecystic fluid, elevated CRP and LFTs, and fibrotic GB as strong predictors of difficult LC. Hutchinson *et al.* (1994) [32] reported that age, male sex, BMI >27.2, prior surgery, GB wall thickening, stones, and CBD dilation increased the likelihood of conversion.

Finally, Schrenk *et al.* (1998) [33] demonstrated that age >65, male sex, obesity, long history of gallstones, recent biliary colic, prior surgery, wall thickness >5 mm, pericholecystic fluid, CBD stones, and elevated bilirubin and LFTs predicted difficult LC with approximately 80% reliability. Collectively, these studies highlight a sequential spectrum of predictors, beginning with baseline patient demographics and comorbidities, progressing through biochemical and ultrasonographic markers of inflammation and biliary obstruction, and culminating in intra-operative challenges such as adhesions, bile spillage, and ductal anomalies, all of which contribute to the risk of difficult laparoscopic cholecystectomy. Overall, when considered sequentially, the evidence demonstrates that difficult LC is a multifactorial process beginning with baseline patient characteristics, progressing through biochemical and radiological markers of inflammation, and culminating in intra-operative findings. Male sex, advanced age, elevated BMI, prior or recurrent acute cholecystitis, gallbladder wall thickening, pericholecystic inflammation, impacted stones, and raised inflammatory markers emerge as the most reproducible predictors. The systematic integration of these variables into validated scoring systems enables reliable preoperative stratification, optimizes surgical planning, and enhances patient counseling and operative safety.

## Discussion:

This narrative review synthesizes current evidence on preoperative scoring systems developed to predict difficult LC. The findings underscore the utility of integrating clinical, radiological and biochemical parameters to enhance surgical planning and patient outcomes. Several studies have identified advanced age as a significant predictor of difficult LC. For instance, a study by Teja *et al.* (2025) reported that patients over 60 years had a higher likelihood of conversion to open surgery, with gallbladder wall thickness and size being pivotal factors in their predictive scoring system [7]. Similarly, other research has corroborated these findings, emphasizing the role of age and gallbladder characteristics in surgical complexity [34]. Radiological assessments, particularly ultrasonography, have proven instrumental in preoperative evaluations. Studies have highlighted that parameters such as gallbladder wall thickness, size and common bile duct diameter are associated with increased operative difficulty [35]. These findings align with the scoring systems developed by Teja *et al.* which incorporated similar ultrasonographic criteria [7]. Biochemical markers also contribute valuable information regarding surgical risk. Elevated white blood cell counts and liver function test abnormalities have been linked to increased operative challenges and conversion rates [34]. The integration of these markers into predictive models can further refine risk stratification. The development of scoring systems, such as those proposed by Teja *et al.* offers a structured approach to predict difficult LC. Their model assigns points based on gallbladder wall thickness and size, providing a quantifiable method to assess surgical risk [7]. This systematic review provides a comprehensive synthesis of studies evaluating predictors of difficult laparoscopic cholecystectomy, encompassing clinical, biochemical and radiological parameters, which allows for a holistic understanding of preoperative risk factors. The inclusion of a large number of studies, rigorous application of PRISMA 2020 guidelines and tabulated comparison of scoring systems enhance transparency and reproducibility, facilitating clinical applicability. Additionally, the chronological presentation of evidence highlights temporal trends in the evolution of predictive models. However, limitations include heterogeneity among the included studies in terms of sample size, study design and scoring criteria, which may affect the generalizability of findings. The review is also constrained by reliance on published literature only, introducing potential publication bias and the lack of meta-analytic synthesis prevents quantification of effect sizes or pooled predictive accuracy. Furthermore, newer predictive models published after the literature search may not have been captured. Such tools can aid clinicians in preoperative counseling and decision-making.

## Conclusion:

Difficult laparoscopic cholecystectomy can be accurately predicted using clinical, biochemical and radiological factors. Key predictors such as age, gender, acute cholecystitis history, BMI and gallbladder conditions aid in identifying high-risk patients. Structured preoperative assessments enhance surgical planning, reducing complications and conversion rates.
